# Lignin and Its Pathway-Associated Phytoalexins Modulate Plant Defense against Fungi

**DOI:** 10.3390/jof9010052

**Published:** 2022-12-29

**Authors:** Vincent Ninkuu, Jianpei Yan, Zenchao Fu, Tengfeng Yang, James Ziemah, Matthias S. Ullrich, Nikolai Kuhnert, Hongmei Zeng

**Affiliations:** 1 State Key Laboratory for Biology of Plant Diseases and Insect Pests, Institute of Plant Protection, Chinese Academy of Agricultural Sciences (CAAS), Beijing 100193, China; 2 Department of Life Sciences and Chemistry, Jacobs University, College Ring 1, 28759 Bremen, Germany

**Keywords:** lignin, pathway enzymes, defense metabolites, plant immunity

## Abstract

Fungi infections cause approximately 60–70% yield loss through diseases such as rice blast, powdery mildew, Fusarium rot, downy mildew, etc. Plants naturally respond to these infections by eliciting an array of protective metabolites to confer physical or chemical protection. Among plant metabolites, lignin, a phenolic compound, thickens the middle lamella and the secondary cell walls of plants to curtail fungi infection. The biosynthesis of monolignols (lignin monomers) is regulated by genes whose transcript abundance significantly improves plant defense against fungi. The catalytic activities of lignin biosynthetic enzymes also contribute to the accumulation of other defense compounds. Recent advances focus on modifying the lignin pathway to enhance plant growth and defense against pathogens. This review presents an overview of monolignol regulatory genes and their contributions to fungi immunity, as reported over the last five years. This review expands the frontiers in lignin pathway engineering to enhance plant defense.

## 1. Introduction

Plants are relentlessly exposed to pest and pathogen attacks. However, their sessile nature is naturally compensated for by synthesizing stress-responsive metabolites to overcome these attacks. Whereas many ribosome-inactivating proteins are reported to render pathogens proteins inactive to confer immunity in plants [1,2], the cell wall’s dynamic and intricate nature provides the first line of defense and environmental cues [3,4]. Several metabolites, including lignin, cellulose, and pectin, contribute to cell wall integrity (CWI) [5]. Lignification, as an integral component of CWI, crucially enhances the two layers of plant innate immunity: pathogen-associated molecular patterns (PAMPs)-triggered immunity (PTI) and effector-triggered immunity (ETI) [6]. While PTI uses pattern recognition receptors to monitor PAMPs on the cellular surface, ETI relies on nucleotide-binding domain leucine-rich repeat receptors to recognize pathogen effectors inside the cell [7]. 

The phenylpropanoid pathway is the metabolic hub of plants and produces approximately 8000 metabolites that enhance robust antagonistic and informative interactions between plants and their environments [8]. Recent insights underscore molecular factors regulating phenylpropanoids’ metabolism orchestrated by a network of enzyme cascades, including; ligases, oxygenases, transferases, and oxidoreductases [9,10,11]. These enzymes influence the chemical modification of metabolic skeletons through glycosylation, acylation, hydroxylation, and methylation. Therefore, the diversity of phenylpropanoid-derived metabolites depends on them [12].

Lignin production is an off-shoot of the phenylpropanoid pathway. *PHENYLALANINE* (*PAL*) is synthesized via the chorismate pathway in plastids and released into the cytosol. It then catalyzes the first of three steps in the general phenylpropanoids pathway. Other regulators of monolignol biosynthesis include C4H, 4CL, the soluble C3H, HCT, CCoAOMTs, COMTs, F5H, CAD, and CCR. Peroxidases and laccases (*PRX/LACs*) encode monolignol polymerization into intracellular spaces of the cell wall [13,14,15,16]. Knowledge of the regulatory mechanism of monolignol biosynthesis continues to expand. Previously, *p*-hydroxyphenyl (H), guaiacyl (G), and syringyl (S) lignin were the only known lignin monomers. Recent studies have reported that catechyl (C) and *5*-*Hydroxy*-guaiacyl (5H) monomers contribute to lignin polymerization in some plant species [17,18,19,20]. A total of 11 lignin family enzymes and 24 metabolites are currently associated with the lignin pathway [17,18,19,20]. However, these metabolites are often credited for their direct involvement in plant defense, whereas the enzymes regulating their accumulation remain in the shadows.

Studies have shown that mutant phenotypes of lignin regulators either shut down or severely impair the molecular switches for lignin and other metabolite accumulation. Compromised lignin metabolism affects plant defense against fungi and overall yield output. Therefore, exploiting the potential contribution of lignin and pathway-related metabolites could contribute to plant growth and yield. Over the last five years, many insightful reports have been published regarding lignin biosynthesis and plant defense. Although reviews on phenylpropanoid biosynthesis have recently been published, none has focused on the individual enzymes that regulate lignin formation and their roles in fungal defense. This review provides an overview of lignin pathway enzymes’ contributions to defense lignification and other pathway-associated metabolic accumulation. 

## 2. A Brief Overview of Monolignols Biosynthesis and Lignification

**Figure 1:**
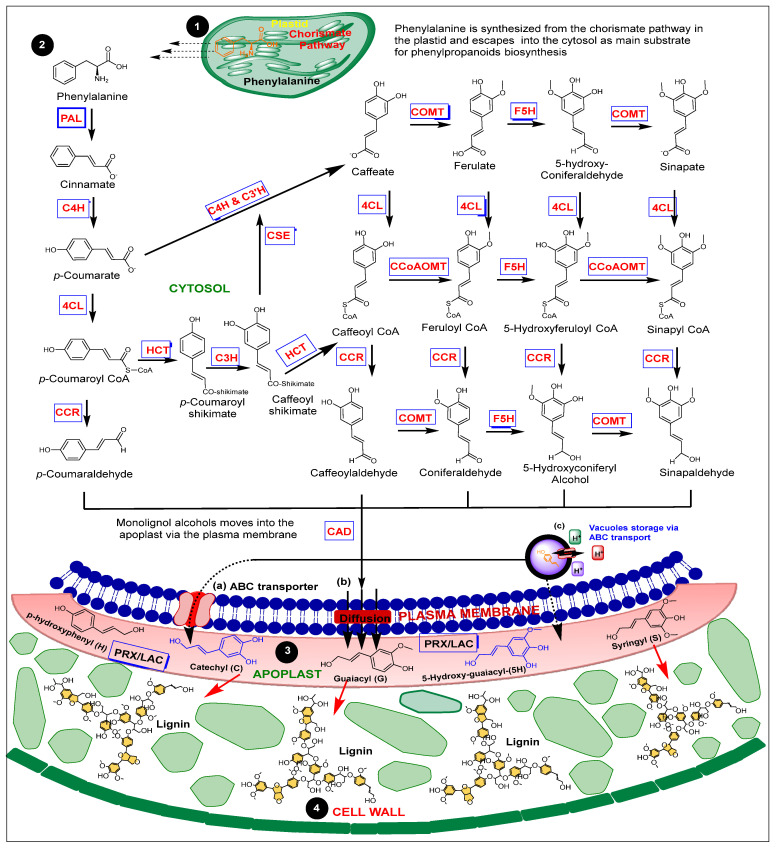
Monolignols biosynthesis and polymerization. The various enzymes leading to monolignol formation are based on current understanding: traditional monolignols (black) and recently discovered monolignols in some plant species (blue). Stage 1: Phenylalanine escapes from the chorismate pathway in the plastid into the cytosol. Stage 2: Enzymatic activities that occur prior to monolignol formation. Stage 3: Monolignols are transported into the apoplast. Stage 4: *PRX/LAC* encodes monolignol polymerization into lignin. Lignin fills up intercellular voids to enhance cell wall rigidity. Proposed mechanism of monolignol transport: (**a**) ABC transporters mediate active trafficking of monolignols. (**b**) Trans-membrane diffusion of monolignols/channels-facilitated membrane transport. (**c**) ABC transporters channel monolignol glycoside into vacuoles for release at cell death.

Even though phenylalanine is not a primary precursor to lignin biosynthesis, it initiates the first of three reaction steps to pave the way for lignin production [18,21]. Research advances have discovered eleven enzymes involved in monolignol production and polymerization (Figure 1) [22]. The functions of each enzyme in the lignin pathway and its defense mechanisms are discussed in this review. In addition, adenosine and cytosine (AC element) enrich DNA motifs to promote lignin synthesis. *MYELOBLASTOSIS* (*MYB*) viral oncogene homolog transcription factors possess a rich AC motif that regulates lignin pathway genes, although they do not actively participate in the biosynthesis process. For example, *MYB46* and its *MYB83* homolog regulate phenylpropanoids and lignin biosynthesis [23,24]. In *Arabidopsis thaliana*, *MYB15* activates *PAL*, *C4H*, *4CL*, *HCT*, *C3H*, *COMT*, and *CAD* to enhance lignin accumulation during defense against *Pseudomonas syringae* DC3000 (*AvrRpm1*) [25].

Uncompromised pathogens penetrate the apoplast or cytosol through the intercellular voids within the cell wall. Lignification is an essential process that resists the entrance of these pathogens by lignin deposition in the voids via Golgi-mediated vesicles in the cell membrane, as recently proposed [26]. Lignification promotes the chemical alteration of pathogen-secreted cell-wall-degrading enzymes to boost toxin diffusion resistance [6]. Some reports suggest lignification disrupts these pathogen-degrading enzymes and restricts pathogens’ mobility in infected cells from infecting new cells [6]. Lignin and callose deposition are also reported to block fungi haustoria from the cell wall [27].

### 2.1. PHENYLALANINE AMMONIA-LYASE (PAL)

*PAL* initiates the general phenylpropanoid pathway reaction by catalyzing the deamination of L-phenylalanine to trans-cinnamic acid and ammonium [16,28]. This process paves the way for several enzymatic activities to produce an enormous array of secondary metabolites, such as lignin, lignan, chlorogenic acid, SA, and stilbene [29]. *PAL* accumulation is linked to defense mediation against pathogens and pests, even though the mechanism by which they execute these activities is elusive. For example, overexpression (OE) and RNA interference (RNAi) enhanced the expression of soybean *GmPAL2.1* against *Phytophthora sojae* infection. The highly expressed *PAL* induced the accumulation of daidzein, glyceollin, genistein, and salicylic acid (SA) to mediate defense against *P. sojae* [30]. The rice genome has nine *PAL* genes. Eight induce resistance against *Magnaporthe oryzae* infection. In addition, *Rhizoctonia solani* stimulates quantitative trait loci for resistance in seven *OsPAL* genes [31].

The *Brachypodium distachyon* (purple false brome), *PAL1*, was also identified to induce lignin, SA, cinnamic acid, and fatty acid accumulation in defense against the panicum mosaic virus. However, RNAi-mediated knockdown of *BdPAL1* enhances *panicum mosaic virus* pathogenicity [32]. *PAL* is also reported to induce lignin and cinnamaldehyde accumulation against *P. capsici* infection in black pepper and trans-cinnamic acid defense against *Xoo* [33]. The *PAL* gene family encodes the production of defense metabolites irrespective of the reaction direction (forward or reverse) and, therefore, are candidate genes for genetic engineering. However, their substratum specificity, catalytic, and protein-wide mechanisms remain elusive, hindering their engineering potential. Table 1 summarizes the current reported role of lignin regulatory genes in fungi immunity in plants.

### 2.2. CINNAMATE 4-HYDROXYLASE (C4H)

The *C4H* is a member of the CYP73A class of P450-associated monooxygenase family proteins that encodes the hydroxylation of *p*-coumaric acid from cinnamic acid. *C4H* activities promote cell wall lignification and biosynthesis of other plant defense metabolites [34,35,36]. The soybean *C4H1* gene is highly responsive to pathogens and encodes defense lignification against *P. sojae*. Whereas the *gmc4h1*-mutant plants are highly susceptible to *P. sojae*, the OE-*GmC4H1* lines in *N. benthamiana* significantly accumulated lignin for immunity induction [34]. *C4H1*, *C4H2*, and *C4H3* expression vary from tissue to tissue in *Pyrus bretschneideri* (pear plant) [35]. Transcripts of *C4H1* and *C4H3* defensively accumulate lignin and robust cell walls in *Arabidopsis* plants overexpressing these genes [35]. A related study reports that *OsC4H* complements pathogenesis and antioxidant-related genes to activate defense against pests [36]. Pathway perturbations can also externally or internally influence biological functions, such as metabolic changes [37]. A reprogrammed phenylpropanoid pathway by piperonylic acid (PA)-mediated inhibition of *C4H* triggers systematic resistance against a broad spectrum of pathogens [38]. The C4H-inhibited *Solanum lycopersicum* (tomato) increased flavonoid production with enhanced immune signaling, cell wall modification, phenolic compounds, and SA accumulation [38]. Elicitor proteins and transcription factors have also been reported to activate C4H defense against fungi (Table 1).

### 2.3. 4-COUMARATE-COA –COENZYME A LIGASE (4CL)

The *4CL* protein distributes the flux among different metabolic pathways. It is the precursor for downstream biosynthesis of other metabolites, such as stilbenes and flavonoids, and also encodes the esterification of *p*-coumaroyl CoA to *p*-coumaric acid for lignin production [9]. A *Fraxinus mandshurica* OE-*4CL2* in tobacco plants enhanced lignin accumulation but inhibited hemicellulose production. This resulted in a 250 % increase in coniferyl alcohol levels, fortifying cell wall and xylem cell layer thickness. Overexpression lines in soybean significantly induced resistance against *P. sojae* by accumulating daidzein, genistein, and glyceollins. The *Fm4CL2* ortholog from *Dryopteris fragrans* (*Df4CL2*), transformed into tobacco via an *Agrobacterium tumefacient*-mediated system, increased lignin and flavonoids concentration, further suggesting *4CL* could play a crucial role in cell-wall-mediated defense [39,40,41].

Transcription factors activate the expression of phenylpropanoid genes. The peach *WRKY70* activates *4CL* and *PAL* promoters to elevate total phenolics, flavonoids, and lignin biosynthesis against a rot initiation fungus, *Rhizopus stolonifer* [42]. *WRKY*, *MYB*, and *bHLH* transcription factors can also switch on lignin biosynthetic genes (*4CL*, *PAL*) in *Pinus strobus* (eastern white pine) after perceiving nematode (*Bursaphelencus xylophilus*)-inflicted injuries [43]. The high expression of *4CL* and *PAL* induces stilbenoids, pinosylvin monomethyl, and monoethyl ethers elicitation to mediate plant defense [43]. In related findings involving *Botrytis cinerea* (gray mold) infection in blueberry fruits, methyl jasmonate (MeJA) treatment restrained the decaying success of gray mold in the fruits through *4CL*-, *C4H*-, and *PAL*-induced production of NO, H_2_O_2_, phenolic, and flavonoid [44].

**Table 1:** Contrition of monolignol biosynthetic regulators to fungal defense.

No.	Gene/Protein	Plant	Research Strategy	Results Obtained	Metabolites	References
1	*MdMRLK2*	*Malus mellana*	Overexpression MdMRLK2 cucurbits	Suppressed PAL, β-1,3- glucanase, chitinase	Inhibited polyphenol synthesis	[45]
2	*AtERF114*	*A. thaliana*	RNAseq, overexpression, knockout	ERF114 activates PAL1 to mediate *P. syringae pv tomato* (*Pst*) defense	Lignin and SA	[46]
3	*PAL1*, *4CL5*, *MYB308*	*Prunus persica*	Overexpression MYB308	PAL1 and 4CL5 enhanced expression-induced resistance against *R*. *stolonifer*	Chlorogenic, gallic acid, and rutin	[47]
4	*POX*, *PAL*	*Zea mays*	Inoculated maize genotypes (P1630H, AG3700, SCS156 Colorado and 30K75Y) with *Bipolaris maydis*	POX, PAL transcript abundance conferred resistance to *B. maydis* in AG3700	phenolic and flavonoids	[48]
5	*PAL*, *POD*	* Nicotiana tobaccum *	Thiamine (vitamin B1, VB1) treatment	Increased *PAL*, *POD*, H_2_O_2_ accumulation, and catalase and peroxidase activities conferred resistance against *Phytophthora nicotianae*	-	[49]
6	*WRKY1*	*Ocimum sanctum*, *A. thaliana*	Overexpression and VIGS OF *WRKY1*	*WRKY1* regulates *PAL* and *C4H* resistance to *P. syringae* pv. tomato Pst DC3000	-	[50]
7	*PAL*	*Phoenix dactylifera*	Alginate extract from *Bifurcaria bifurcata* was tested agaisnt *F. oxysporum*	Alginate treatment triggered *PAL* expression against *F. oxysporum* f. sp. Albedinis	-	[51]
8	* C4H * , *CAD*, *POD*	*Prunus persica*	RNAseq, transient overexpression of *PpMYB306*	* P. guilliermondii * inhibits *PpMYB306* repressed lignin genes in peach after * R. stolonifer * infection.	Inhibited lignin content	[52]
9	*C4H*, *COMT*, *BAK1*, *WRKY5*	*Olea europaea*	Analysis of defense mechanism of tolerant and susceptible olive cultivars to *V. dahliae*	*V. dahlia*-tolerant cultivar significantly accumulated root lignin after *V. dahlia* inoculation	Lignin	[53]
10	*PALs*, *Cl4Cls*, *CYP73A*, *CCR ClHCTs*	*Citrullus lanatus*	RNA-Seq of resistant ZXG1755 and susceptible ZXG1996 lines inoculated with powdery mildew during the early seedling stage	Hormonal, lignin and peroxidase transcripts were significantly expressed	Lignin and phytohormone biosynthesis	[54]
11	* ScAPD1- * like	* Syntrichia caninervis *	Overexpression of *ScAPD1*-like in *Arabidopsis* and *S. caninervis*	Defense against *V. dahliae*, decreased ROS synthesis, improved ROS scavenging activity, enhanced lignin (*PAL*, *C4H*) transcripts	High lignin accumulation	[55]
12	*Hrip1*	*Oryza sativa*	RNAseq and metabolic analysis of Hrip1-treated rice leaves	Hrip1 mediates defense against rice blast fungi by activating *PAL*, *C4H*, *4CL*, *HCT*, *C3H*, *COMT*, *CAD*, *PRX*, diterpene synthases (*CPS2*, *-4*, *KSL4*, *5*, *-6*, *-7*, *10*, cytochromes (CYP71Z, CYP7M, momilactone synthases), benzoxazinoids biosynthetic genes (BX1-BX7)	Lignin, diterpenoids	[56]
13	* WRKY * , *PAL*, *CHI*	* Vigna angularies *	Transcriptome and histological analysis of *Vigna angularies* against *Uromyces vignae*	PRRs recognize *U. vignae* invasion and activities *PAL*, *WRKY*, *CHI* defense	-	[57]
14	*CAD35*, *CAD45*, *CAD43*	*G. hirsutum*	VIGS and overexpression of GhCAD35, *GhCAD45*, or *GhCAD43*	VIGS of CAD genes inhibited S-lignin production, ultimately affecting the syringyl/guaiacyl (S/G) ratio, while OE-lines enhanced *V. dahliae* defense	Lignin, SA	[58]
15	* PAL * , *4CL*, * COMT * , *CAD POX*	*Panax notoginseng*	Transcriptomic and proteomic technologies	*Alternaria panax* inoculation activated *PAL*, 4CL, *COMT*, *CAD*, *POX* expression	Lignin	[59]
16	* PAL *	* Cajanus cajan *	Metabolic analysis	*Fusarium udum* induced the expression of lignin-related transcripts and enzyme activities for lignin and phenolic acids accumulation	Phenolics, lignin	[60]
17	*COMT*, *PRX*, *CAD*, *HCT*	* Malus domestica *	Comparative RNA-seq analysis	*Malus domestica* inoculated with Fpmd MR5 induced the expression of several lignin genes, antimicrobial and antioxidants genes	-	[61]
18	* COMT1 *	*Triticum aestivum*	Transcription profiling of genes involved in *Triticum aestivum- Puccinia striiformis* interaction	* COMT1 * was highly expressed in response to *Puccinia striiformis* inoculation	-	[62]
19	*GhODO1* *Gh4CL1*, *GhCAD3*	*G. hirsutum*	*GhODO1*-GFP transient expression in onion, qPCR, lignin quantification	* GhODO1 * binds to * Gh4CL1 * and * GhCAD3 * promoters to activate lignin-enhanced resistance to * V. dahliae *	Lignin, JA	[63]
20	* LCC24 * , *ROMT*, *LCC24*,	* Elaeis guineensis *	Analysis of oil palm defense against *Ganoderma boninense* inoculation, qPCR, and metabolic analysis	oil palm cultivar, C08 exhibited high resistance by activating *Ganoderma boninense*	SA, lignin	[64]
21	Xylogen-like *arabinogalactan protein1* and -*2*	*Capsicum annuum*	Genome-wide studies, phylogenetics, and VIGS analysis	Enhanced expression of lignin genes and lignin accumulation in pepper stem.	Lignin	[65]
22	Ammonia-lysases (ALs)	*B. distachylon*	Proteomics, RNAi knockdown, metabolic analysis	Ammonia-lysases performed a central role in carbon allocation for lignin accumulation and shikimate ester does not contribute to lignin synthesis in *B. distachylon*	Lignin	[66]

A virus-induced gene silencing (VIGS) of *4CL30* in cotton compromised lignin and flavonoid accumulation but increased caffeic and ferulic acid levels to confer immunity against *Verticilia dahlia* [67]. The central position of flux distribution showed that *4CL* is an essential enzyme in downstream defense modulation (Table 1) and could play a critical role in lignin pathway engineering.

### 2.4. HYDROXYCINNAMOYL TRANSFERASE (HCT)

The *HCT* distributes the mass flux among C-, G-, 5H, and S-lignin. It also forms *p*-coumaroyl shikimic acid from *p*-coumaroyl CoA and then reversely encodes caffeoyl shikimate conversion to caffeoyl CoA [68,69]. However, the latter process is being questioned for possible redundancy. In *O. sativa*, the negative regulation of cell death elicitation mediated by the *APIP5* transcriptional factor that binds to *OsPHCT4* is mitigated by *APIP5*-RNAi [70]. This process frees up the activation of tryptamine *HCTs* (*OsTBT1* and *OsTBT2*) and tyramine *HCTs* (*OsTHT1* and *OsTHT2*) to enhance immunity against *M. oryzae* through lignin and phenolamide accumulation [70]. *Populus trichocarpa WRKY* transcription factor regulates *HCT2* to mediate defense against *Sphaerulina musiva* [71], while *MYB15* turns on monolignol synthetic genes, including *HCT*, for lignin-mediated ETI [25]. *Populus tomentosa Carr PtoHCT1* also relies on caffeoyl-CoA and shikimic acid substrates to synthesize caffeoyl shikimate. *PoptrHCT1* and *-2* from *Populus trichocarpa*, a close relative of *P. tomentosa*, contribute to plant defense [20]. Similar investigations involving *HCT* defense against fungi have been reported (Table 1).

### 2.5. CAFFEOYL SHIKIMATE ESTERASE (CSE)

*CSE* catalyzes the direct conversion of caffeoyl shikimate to caffeate acid. The reverse catalytic activity of *HCT* in converting caffeoyl shikimate to caffeoyl-CoA has raised controversy upon *CSE* discovery [72], suggesting this process could be redundant in the lignin pathway. Even though there is no established consensus, available reports suggest *CSE* could be more efficient than *HCT* in lignin biosynthesis [73]. A few recent reports have elucidated the function of *CSE* in lignin production. However, no distinct bioassay demonstrating the defense function of this enzyme in vitro via the higher lignin content has been reported in the last five years. *CSE* from a hybrid Populus significantly encodes lignin accumulation [74]. Moreover, its OE-*PbCSE1* lines in pea fruits increased lignin content in the stem [75], while its mutant lines decreased lignin production [76].

### 2.6. CAFFEOYL-COENZYME A 3-O-METHYLTRANSFERASE (CCoAOMTs) and CAFFEIC ACID 3-O-METHYLTRANSFERASE (COMTs)

*CCoAOMTs* and *COMTs* catalyze the hydroxyl-methylations in the phenylpropanoids pathway [77,78], making them integral members of monolignol, coumarins, caffeic, and sinapic acids biosynthesis with amplified roles in plant defense [13,14,15,16]. For example, the OE-*CCoAOMT* lines in *Paeonia ostii* (tree *peony*) and *Camellia sinensis* (tea plant) induce lignin production [13,14,15,16] for potential defense roles besides ROS scavenging and drought tolerance. *Activated LrCCoAOMT* from *Lilium regale* (*royal lily*) is highly responsive to *B. cinerea* and induces SA signaling. The OE-*LrCCoAOMT* in *Arabidopsis* accumulates more lignin in the vascular tissue against *B. cinerea* [79]. Similarly, *Triticum aestivum TaCOMT*-3D participates in defense against *Rhizoctonia cerealis* (Sharp eyespot) infection [80], and its mutants are susceptible to sharp eyespot fungi infection, while OE-*TaCOMT*-3D lines significantly induce defense lignification [80]. A cloned neem *NCOMT* in *Withania somnifera* and *Ocimum* species robustly catalyzed ferulic formation from caffeic acids. Ferulic acid confers additional cell wall rigidity and is a precursor to coniferyl alcohols, sinapic, and curcumin. Therefore, *NCOMT* involvement in these processes could be significant for metabolic engineering against fungi [81].

Sugar cane *ShMYB78* regulates suberin accumulation by activating *COMT* and ketoacyl-CoA synthase (*ShKCS20*) [82]. Suberin is a vital metabolite that provides a physical barrier against pathogens, water loss, and wound healing and could spike interest in possible engineering attempts [82]. CRISPR-Cas9-mediated editing of StCCoAOMT in Russet Burbank potato induces suberin and lignin elicitation to resist *P. infestans* [83]. In addition, the bread wheat plant lignin-induced cell wall thickening was enhanced by *TaCCoAOMT* for Fusarium head blight resistance [84].

### 2.7. FERULATE 5-HYDROXYLASE (F5H)

The *F5H* is the third P450-dependent protein that regulates lignin biosynthesis. It catalyzes S-monolignol from G-monolignol through 5-hydroxylation of coniferaldehyde and coniferyl alcohols [85,86]. The role of *F5H* in lignin production is proposed to be thwarted by microRNA from *Bacopa monnieri* (*Bm-miR172c-5p*) which cleaves *F5H* and interferes with lignin elicitation [87]. Seedlings of OE-*Bm-miR172c-5p* rendered lignin-induced secondary cell wall thickening redundant under drought-stress conditions, but overexpressing the mimic target, eTMs, restored lignification and secondary cell wall thickening [87]. Hence, *Bm-miR172c-5p* maintains *B. monnieri* native phenotype under different environmental conditions. The OE-*PtoF5H* lines in *P. tomenta* mediate the proportional enhancement of S-monolignol [85].

Monolignol ratio is also reported to influence biomass recalcitrance and plant disease resistance. A CRISPR/Cas9-mediated knockout of four *F5H* (*ko-7*) genes from *Brassica napus* (oilseed rape) reduced the syringyl:guaiacyl monolignol ratio (S: G). The *ko-7* mutant developed resistance against pathogenic *Sclerotinia sclerotiorum* (stem rot) through cell wall fortification [86]. *F5H* also confers immunity against parasitic plants. *Striga hermonthica* (purple witchweed) infects rice, maize, and sugar cane in Asia and Sub-Saharan Africa. *Striga*-resistant Nipponbare and susceptible Koshihikari cultivars preferentially accumulate lignin monomers [88]. The co-expression of *F5H* and *C3H* induced a high stack of H-, G-, and S-lignin to induce rice immunity to *S. hermonthica* [88].

### 2.8. CINNAMOYL COA REDUCTASE (CCR)

CCR encodes the formation of hydroxycinnamaldehydes from hydroxycinnamoyl-CoA, the first committed step in monolignol production. Loss of *CCR* function in angiosperm inhibits lignin accumulation and increases susceptibility to pathogens [89]. *B. nepus CCR1* gene participates in H- and G-lignin synthesis and vascular systems formation, while the *BnCCR2* encodes S-lignin production. OE-*BnCCR* (1 and 2) phenotypes delayed flowering time and resulted in poor leaf and vascular system development [89]. *BnCCR1* and *BnCCR2* increased glucosinolate (GLSs) concentration [89], which could remedy chemical defense against fungi diseases through hormone signaling and pathogen perception [90,91,92].

### 2.9. CINNAMYL ALCOHOL DEHYDROGENASE (CAD)

CAD encodes the NADPH-dependent reduction of various hydroxy-cinnamaldehydes to their respective monolignol alcohols [93]. Rice *CAD2* transcript abundantly accumulates in young seedlings and confers cell-wall-mediated immunity against *Xanthomonas oryzae* pv. *oryzae* (*Xoo*) [93]. Cell wall fortification has been explored to control *Sclerotinia sclerotiorum*. *BnCAD5* and *F5H* induce rapid accumulation of S-lignin against *S. sclerotiorum* infection [26]. A comparative transcriptional analysis in *Manduca sexta* (stem-boring herbivore), *Trichobaris mucorea* (stem borer)-attacked, and healthy wild tobacco *Nicotiana attenuata* implicated *CAD* activity for enhanced lignin deposition in parenchymal cells and pith of the insect-attacked plants. However, *cad* mutants restored the stem-boring ability of the herbivores without inhibiting growth. Ethylene and jasmonate were subsequently identified to signal pith lignification [94].

*Trichoderma harzianum* is a plant fungicide used for foliar application, seeds, and soil treatment to control fungi pathogens. The commercial fungicide 3Tac is developed from *T. harzianum* to control Botrytis, Fusarium, and Penicillium spp. Studies have shown that *T. harzianum* induces immunity in *S. lycopersicum* L (tomato) against RKN, *Meloidogyne incognita* through increased expression of *CAD*, *PAL*, *C4H*, and *CCOMT* for lignin, flavonoids, and phenols accumulation against *M. incognita* [95]. The transformation of another *CAD2* gene from *Pyrus pyrifolia* (pear) into a tomato plant via an *Agrobacterium*-mediated system defensibly accumulated lignin in leaves, stems, and fruits [96].

### 2.10. PEROXIDASES and LACCASES (PRX and LACs)

Plant cell wall lignification is catalyzed by class III peroxidase (*PRX*) and laccase (*LACs*) enzymes [97,98] for defense modulation and breakdown of hydrogen peroxides in the cytosol and chloroplast [99]. An apoplast *CsPRX25* protein in *Citrus sinensis* induces cell wall lignification to mediate defense against pathogens [100]. Blossom-end rot also induces ROS, H_2_O_2_, and lignin accumulation. According to Reitz & Mitcham, enhanced expression of *PRXs* in blossom-end rot-infected tomatoes participates in defense lignification [101]. In addition, two PRX genes (VlPRX21 and VlPRX35) in the grapevine are involved in trans-resveratrol conversion to δ-vinifera and could be essential genes for δ-viniferin engineering for enhanced fungal defense in plants [102]. Histochemical analysis showed the localization of lignin in the xylem cell wall was linked to *DcPRX30*, *DcPRX32*, and *DcPRX62* activities in the taproot epidermal zones of carrots, leading defense lignification [103].

A VIGS *talac4* mutant in QTL-Fhb1 of wheat NILs increases the plant susceptibility to *F. graminearum* infection with low lignin elicitation compared with the wild type [104]. In addition to lignin, coniferin, coumarins (isopimpinellin), and 5,6,7-trimethoxycoumarin defensibly accumulated against *F. graminearum.* Docosanoic acid and 1-O-Vanilloyl-beta-D-glucose also provided complimentary protection against *F. graminearum* [104]. PRXs also induce defense accumulation of NADPH oxidases and apoplastic ROS. For instance, Arabidopsis *PRX33* and *PX34* knockdown mutants reduced H_2_O_2_ content in response to PAMP treatments and PAMP-induced protein expression [105].

## 3. Phytoalexins Associated with the Lignin Pathway Enzymes

**Figure 2:**
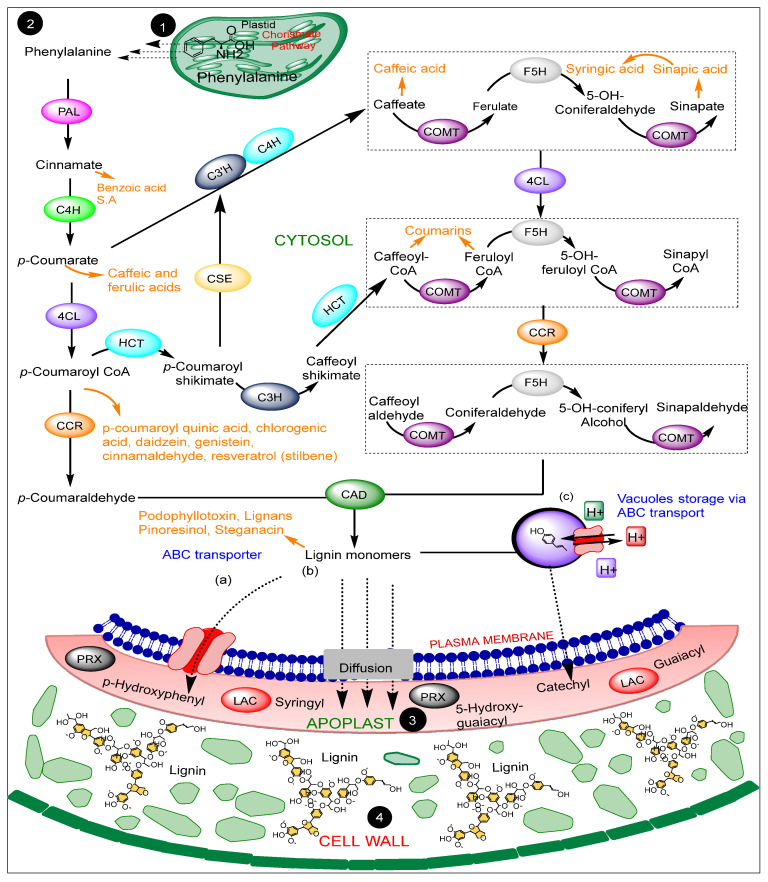
Defense metabolites associated with the lignin pathway and encoded by the pathway enzymes Defense metabolites are illustrated in orange, highlighting their biosynthesis routes in the pathway. The distribution of the metabolites is based on the current knowledge of their biosynthesis. Steps 1, 2, 3, and 4 are the same as in Figure 1. The proposed monolignol transport mechanisms (**a**–**c**) are also the same as in Figure 1.

Apart from lignin being the final product and most crucial metabolite in this pathway, other antifungal defense metabolites accumulate along the same path (Figure 2). Current advances link coumarin accumulation to the catalytic activities leading to *p*-coumaryl CoA formation. Therefore, *PAL*, *4CL*, and *HCT* play a role in coumarin biosynthesis. The feruloyl-CoA formation from the *p*-coumaryl CoA precursor forms the committed step for coumarin accumulation with the involvement of the *CCoAOMT* enzyme. Moreover, iron-assisted hydroxylation of cinnamate, *p*-coumarate, caffeate, and ferulate also accumulates simple coumarins. Umbelliferone, esculetin, and scopoletin are simple coumarins whose biosynthesis follows this route [106,107,108]. Coumarins have generally been reported as plant microbiome regulators, principally regulating three crucial activities: nutrient improvement, pathogen inhibition, and abiotic stress tolerance [107,109,110].

Stilbenes are also phenolic phytoalexins whose accumulation is also associated with the lignin pathway regulators. They are unique for their C_6_-C_2_-C_6_ carbon skeleton [111]. *PAL*, *C4H*, and *4CL* activities in the phenylpropanoid pathway leading to *p*-coumaroyl-CoA formation, as elaborated in Figure 1 and Figure 2, generate an active intermediate for trans-resveratrol production. Finally, stilbene synthase (STS) catalyzes the conversion of *p*-coumaryl-CoA to the stilbene skeleton by initially converting *p*-coumaroyl-CoA and a three-unit malonyl-CoA to trans-resveratrol. STS also converts cinnamoyl-CoA to *trans*-pinosylvin. Moreover, resveratrol-*O*-methyl transferase is enhanced by *VvMYB14* and *VvMYB15* for stilbene production [112,113,114]. The defense involvement of stilbene against fungi and viral diseases are recently reported [115,116,117].

Furthermore, caffeic acid is the precursor to ferulic acid. Both share the same route from the phenylalanine precursor through the 4-hydroxycinnamic acid precursor leading to the formation of caffeic acid. Caffeic acid subsequently becomes the precursor to ferulic acid biosynthesis in plants, regulated by *COMT* enzymes in the lignin pathway. As well as lignin and lignan biosynthesis intermediates, caffeic, ferulic, and dihydro ferulic acids are lignocellulose compounds. They induce cell wall stiffness by crosslinking with lignin and other polysaccharides [118,119]. *PAL*, *C4H*, and *4CL* chronologically catalyze the formation of the coumaroyl CoA precursor for downstream biosynthesis of daidzein and genistein through the initial enzymatic activities of chalcone synthase (CHS). Daidzein and genistein accumulation is induced by fungi, bacterial, and viral infections [120,121].

**Figure 3:**
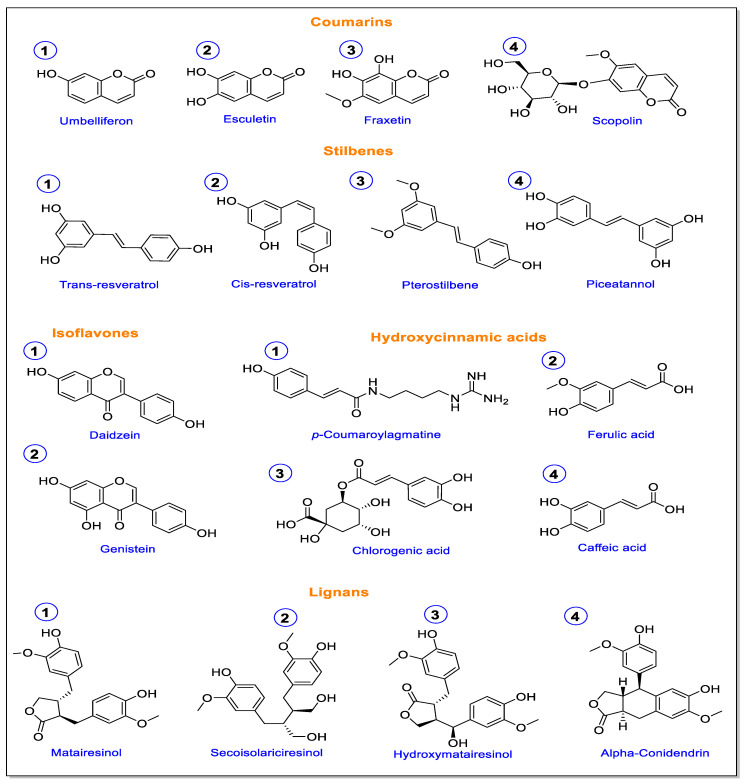
Non-lignin defense metabolites associated with the lignin biosynthetic pathway. This Figure was created using ChemDraw Professional, version 20.0.41, and the structures were analyzed and confirmed using https://pubchem.ncbi.nlm.nih.gov/ structure inquiry (accessed on 16 December 2022).

Lignans are vital physiological, developmental, and ecological plant metabolites. They are formed by coupling reactions of monolignols and defend against herbivores and pathogens [122]. Plants’ dirigent protein crucially regulates the initial coupling reactions that form lignans. *PINORESINOL-LARICIRESINOL REDUCTASES* (*PLR*) then encode successive reduction reactions to form lariciresinol and secoisolariciresinol from pinoresinol. A soybean dirigent protein (*GmDIR22*) was identified to regulate coniferyl alcohol coupling into lignan (+)-pinoresinol to restrict *P. sojae* hyphal growth. An enhanced concentration of yatein was detected in the roots and leaves of mycorrhizal plants in conferring resistance against *B. cinerea* infections [123]. The chemical structures of lignin pathway-associated phytoalexins shown in Figure 3.

## 4. Missing Links in the Lignin Research, Prospects, and Conclusions

The lignin pathway is a crucial vehicle for plant information and communication interactions with their environment and a source of bioactive compounds for plant defense. As a result, a thorough understanding of the pathway enzymes and their interactions will contribute significantly to the beneficial exploits of fungi defense tradeoffs. Enormous literature on key genes regulating lignin biosynthesis and their activities abound. This review dissected a plethora of them, including some defense metabolites that accumulate along the lignin pathway. Engineering these candidate genes in food crops could promote disease resistance to enhance crop yield. However, there are several unanswered questions on lignin metabolism that could facilitate its engineering processes. The shikimate pathway involves seven enzymatic steps to form folates and aromatic amino acids in plants, including phenylalanine. This process exclusively occurs in the plastid, and shikimate provides the required substrate for phenylalanine formation. The mechanism involved in shikimate transition into the cytosol for lignin biosynthesis is currently unknown. In addition, CSE directly converts caffeoyl shikimate to caffeic acid, a shorter route to monolignol biosynthesis. It is also unclear if this process renders the HCT role in reverse reaction redundant. Further identifying the most efficient route between the two could enhance lignin genetic manipulations to address pathogen defense. More lignin monomers are identified in some plant species. Intriguingly, current reports only focused on the dimerization and polymerization reactions that form lignan and lignin, respectively, but the key functions of the individual monolignols relative to plant defense are unknown. In a nutshell, addressing these gaps will improve the attempts of lignin pathway engineering to enhance plant defense against fungi.

## Data Availability

Not applicable.
